# (*E*)-3-(1-Naphthyl­amino)­methyl­ene-(+)-camphor

**DOI:** 10.1107/S1600536810052487

**Published:** 2010-12-18

**Authors:** Jesús Pastrán, Emilio Ineichen, Giuseppe Agrifoglio, Anthony Linden, Romano Dorta

**Affiliations:** a Departamento de Química, Universidad Simón Bolívar, Caracas 1080A, Venezuela; b Institute of Organic Chemistry, University of Zürich, Winterthurerstrasse 190, CH-8057 Zürich, Switzerland

## Abstract

In the crystal structure of the title ketoamine {systematic name: (*E*)-1,7,7-trimethyl-3-[(1-naphthyl­amino)­methyl­idene]bicyclo­[2.2.1]heptan-2-one}, C_21_H_23_NO, there are two independent mol­ecules in the asymmetric unit. Both mol­ecules have an *E* configuration about the alkene function. The main conformational difference between the mol­ecules is in the orientation of the plane of the naphthyl rings with respect to the camphor fragment. The torsion angle about the enamine C—N bond is 21.3 (7)° for mol­ecule *A*, but −24.4 (8)° for mol­ecule *B*. Inter­molecular N—H⋯O hydrogen bonds between the amino and ketone groups of adjacent independent mol­ecules sustain the crystal, and the resulting extended chains, containing an alternating sequence of the two independent mol­ecules, run parallel to the [001] direction and can be described by a graph-set motif of *C*_2_^2^(12).

## Related literature

For the conformations of β-ketoamines, see: Zharkova *et al.* (2009 ▶). For chiral camphor-derived β-amino­ketonate ligands, see: Everett & Powers (1970 ▶); Casella *et al.* (1979 ▶). For reactions involving amino­ketonate complexes, see: Hsu, Chang *et al.* (2004 ▶); Hsu, Li *et al.* (2007 ▶); Lai *et al.* (2005 ▶); Pan *et al.* (2008 ▶); Wang *et al.* (2006 ▶). For the coordination chemistry of β-amino­ketonate ligands, see: Lesikar *et al.* (2008 ▶); Sedai *et al.* (2008 ▶). For the synthesis of (+)-hy­droxy­methyl­enecamphor, see: Lintvedt & Fatta (1968 ▶). For related (1-naphthyl­amino)­methyl­ene structures, see: Li *et al.* (2009 ▶); Özek *et al.* (2005 ▶). For graph-set theory, see: Bernstein *et al.* (1995 ▶).
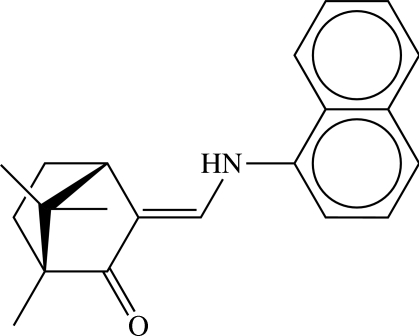


## Experimental

### 

#### Crystal data

C_21_H_23_NO
*M* *_r_* = 305.42
Monoclinic, 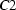

*a* = 23.807 (2) Å
*b* = 11.9688 (12) Å
*c* = 12.0192 (8) Å
β = 95.672 (5)°
*V* = 3408.1 (5) Å^3^
*Z* = 8
Mo *K*α radiation
μ = 0.07 mm^−1^
*T* = 160 K
0.25 × 0.20 × 0.12 mm

#### Data collection

Nonius KappaCCD area-detector diffractometer
21618 measured reflections
3170 independent reflections
2227 reflections with *I* > 2σ(*I*)
*R*_int_ = 0.092

#### Refinement

*R*[*F* ^2^ > 2σ(*F* ^2^)] = 0.059
*wR*(*F* ^2^) = 0.155
*S* = 1.05
3170 reflections
428 parameters
1 restraint
H atoms treated by a mixture of independent and constrained refinement
Δρ_max_ = 0.24 e Å^−3^
Δρ_min_ = −0.17 e Å^−3^

### 

Data collection: *COLLECT* (Nonius, 2000 ▶); cell refinement: *DENZO-SMN* (Otwinowski & Minor, 1997 ▶); data reduction: *DENZO-SMN* and *SCALEPACK* (Otwinowski & Minor, 1997 ▶); program(s) used to solve structure: *SIR92* (Altomare *et al.*, 1994 ▶); program(s) used to refine structure: *SHELXL97* (Sheldrick, 2008 ▶); molecular graphics: *ORTEPII* (Johnson, 1976 ▶); software used to prepare material for publication: *SHELXL97* and *PLATON* (Spek, 2009 ▶).

## Supplementary Material

Crystal structure: contains datablocks I, global. DOI: 10.1107/S1600536810052487/su2235sup1.cif

Structure factors: contains datablocks I. DOI: 10.1107/S1600536810052487/su2235Isup2.hkl

Additional supplementary materials:  crystallographic information; 3D view; checkCIF report

## Figures and Tables

**Table 1:** Hydrogen-bond geometry (Å, °)

*D*—H⋯*A*	*D*—H	H⋯*A*	*D*⋯*A*	*D*—H⋯*A*
N1—H1⋯O2^i^	1.03 (4)	1.93 (4)	2.909 (5)	157 (4)
N2—H2⋯O1	0.83 (5)	2.08 (5)	2.913 (5)	174 (5)

Symmetry code: (i) 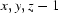.

**Fig. 1.**
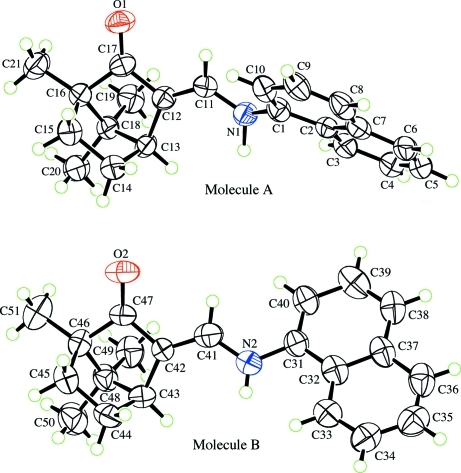
View of molecule A and molecule B of the title compound, showing the atom-labelling scheme. The molecules are oriented independently so as to have the camphor fragments in approximately the same orientation and emphasise the conformational differences between the molecules. Displacement ellipsoids are drawn at the 50% probability level. H atoms are represented by circles of arbitrary size.

**Fig. 2.**
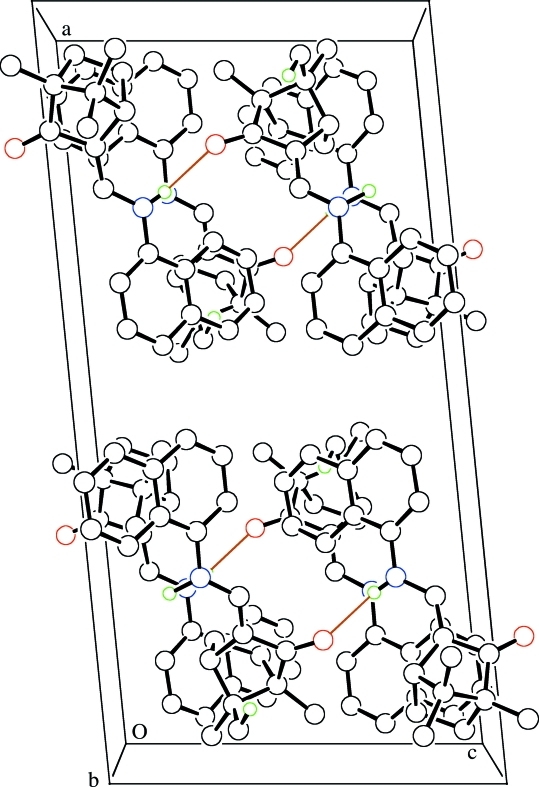
Molecular packing of compound compound projected down the b axis, showing the hydrogen bonding as thin lines [see Table 1 for details]. Hydrogen atoms not involved in hydrogen bonding have been omitted for clarity.

## supplementary crystallographic information

### Comment

β-Ketoamines are the neutral protic form of β-aminoketonate bidentate anionic ligands that have been used in the coordination chemistry of transition and main group metals (Lesikar *et al.*, 2008; Sedai *et al.*, 2008). The electronic and steric dissymmetry of these ligands is easily modified in order to tune the reactivity of the metal centre. β-Aminoketonate complexes have been used effectively in stoichiometric (Hsu, Chang *et al.*, 2004; Hsu, Li *et al.*, 2007) and catalytic processes, such as Suzuki cross-coupling (Lai *et al.*, 2005), polymerization (Wang *et al.*, 2006) and copolymerization (Pan *et al.*, 2008) reactions. Interestingly, there are only a few reports on chiral camphor-derived β-aminoketonate ligands (Everett & Powers, 1970; Casella *et al.*, 1979). Generally, β-ketoamines have *Z* conformations that are stabilized by intramolecular hydrogen bonding (Zharkova *et al.*, 2009). The structure of the title compound was determined in order to confirm the anticipated E conformation about the alkene bond for the major product of the synthesis.

There are two molecules (A and B) of the title compound in the asymmetric unit (Fig. 1). The slightly twisted conformations of the (1-naphthylamino)methylene fragments are similar to that in the structure of 2,2-dimethyl-5-(1-naphthylaminomethylene)-1,3-dioxane-4,6-dione (Li *et al.*, 2009): the absolute values of the torsion angle about the enamine C—N bond for the two structures lie in the narrow range of 21–25°. In contrast, the same group in 2-hydroxy-6-[(1-napthylamino)methylene]cyclohexa-2,4-dien-1-one is almost planar (Özek *et al.*, 2005).

The preference for the E conformation during the synthesis of the title compound may be attributed to the large size of the naphthyl group, whose steric pressure overcomes the competing intramolecular N—H···O hydrogen bonding, which is facilitated in the *Z* conformer. The observed intermolecular N—H···O hydrogen bonds between the amino and keto groups of adjacent independent molecules, which link the molecules into extended chains running parallel to [001]  (Fig. 2), are an additional stabilizing factor of the E conformation. They can be described by a graph-set motif of C^2^_2_(12) [Bernstein *et al.*, 1995].

While the chirality of the (+)-camphor fragment means that both symmetry-independent molecules are of the same enantiomer, it is interesting to note that there is significant pseudo-inversion symmetry in the structure, with 82% of the atoms in one molecule matching closely with those of the inverted structure of the other molecule; the r.m.s. fit of 21 atoms from each molecule is 1.14 Å. Slight in-plane disorder of the naphthyl groups leads to enlarged displacement ellipsoids for some of the atoms of these groups with the direction of elongation being in the naphthyl plane.

### Experimental

The title compound was prepared by refluxing 1-naphthylamine (6.77 g, 37.6 mmol) with (+)-hydroxymethylenecamphor (Lintvedt & Fatta, 1968) (5.92 g, 41.3 mmol) in dry ethanol (200 ml) and formic acid (2.5 ml) for 48 h. After removing the solvent under reduced pressure, the resulting yellow solid was dried *in vacuo* for 4 h. The crude product contained both conformers, which after washing with hexane and HV drying afforded 6.53 g (57%) of the pure (E)-conformer [the (*Z*)-conformer being more soluble in alkanes]. Yellow single crystals suitable for an X-ray analysis were grown from a saturated and filtered ethanol solution that was cooled slowly to 263 K (m.p. 351–353 K). Elemental analysis calculated for C_21_H_23_NO: C 82.58, H 7.59, N 4.59%; found: C 85.26, H 7.99, N 4.61%. NMR and IR Spectroscopic data are available in the archived CIF.

### Refinement

In the final cycles of refinement, in the absence of significant anomalous scattering effects, 2643 Friedel pairs were merged and Δf " set to zero. The enantiomer used in the refinement model was chosen to match the known configuration of the (+)-camphor fragment. The amine H atoms were located in a difference Fourier map and their positions were refined freely with *U*_iso_(H) = 1.2*U*_eq_(N). The C-bound H atoms were placed in geometrically idealized positions and constrained to ride on their parent atoms: C—H = 0.95, 0.98, 1.00 Å, for CH, CH_3_ and CH_2_ H-atoms, respectively, with *U*_iso_(H) = k × *U*_eq_(C), where k = 1.5 for CH_3_ H-atoms and k = 1.2 for all other H-atoms.

### Crystal data

**Table:**

C_21_H_23_NO	*F*(000) = 1312
*M**_r_* = 305.42	*D*_x_ = 1.190 Mg m^−^^3^
Monoclinic, *C*2	Melting point: 352 K
Hall symbol: C 2y	Mo *K*α radiation, λ = 0.71073 Å
*a* = 23.807 (2) Å	Cell parameters from 3158 reflections
*b* = 11.9688 (12) Å	θ = 2.0–25.0°
*c* = 12.0192 (8) Å	µ = 0.07 mm^−^^1^
β = 95.672 (5)°	*T* = 160 K
*V* = 3408.1 (5) Å^3^	Prism, yellow
*Z* = 8	0.25 × 0.20 × 0.12 mm

### Data collection

**Table:**

Nonius KappaCCD area-detector diffractometer	2227 reflections with *I* > 2σ(*I*)
Radiation source: Nonius FR590 sealed tube generator	*R*_int_ = 0.092
horizontally mounted graphite crystal	θ_max_ = 25.0°, θ_min_ = 2.5°
Detector resolution: 9 pixels mm^-1^	*h* = 0→28
ω scans with κ offsets	*k* = 0→14
21618 measured reflections	*l* = −14→14
3170 independent reflections	

### Refinement

**Table:**

Refinement on *F*^2^	Secondary atom site location: difference Fourier map
Least-squares matrix: full	Hydrogen site location: difference Fourier map
*R*[*F*^2^ > 2σ(*F*^2^)] = 0.059	H atoms treated by a mixture of independent and constrained refinement
*wR*(*F*^2^) = 0.155	*w* = 1/[σ^2^(*F*_o_^2^) + (0.0737*P*)^2^ + 1.1311*P*] where *P* = (*F*_o_^2^ + 2*F*_c_^2^)/3
*S* = 1.05	(Δ/σ)_max_ = 0.001
3170 reflections	Δρ_max_ = 0.24 e Å^−^^3^
428 parameters	Δρ_min_ = −0.17 e Å^−^^3^
1 restraint	Extinction correction: *SHELXL97* (Sheldrick, 2008), Fc^*^=kFc[1+0.001xFc^2^λ^3^/sin(2θ)]^-1/4^
Primary atom site location: structure-invariant direct methods	Extinction coefficient: 0.0040 (7)

### Special details

**Table:**

Experimental. Solvent used: EtOH. Cooling Device: Oxford Cryosystems Cryostream 700. Crystal mount: glued on a glass fibre. Mosaicity: 1.498 (4)°. Frames collected: 273. Seconds exposure per frame: 88. Degrees rotation per frame: 1.4. Crystal-Detector distance: 30.0 mm.Spectroscopic data:^1^H-NMR (400 MHz, CDCl_3_): δ 10.77 (d, J = 12.0 Hz, 1H), 8.09 (d, J = 8.0 Hz, 1H), 7.80 (d, J = 12.0 Hz, 1H), 7.54–7.45 (m, 3H), 7.40–7.36 (t, 1H), 7.20 (d, J = 12.0 Hz, 1H), 7.11 (d, J = 8.0 Hz, 1H), 2.53–2.52 (d, J = 4.0 Hz, 1H), 2.11–2.05 (m, 1H), 1.73–1.66 (m, 1H), 1.49–1.41 (m, 2H), 1.03 (s, 3H), 0.94 (s,3*H*), 0.89 (s, 3H); ^13^C {^1^H}-NMR (101 MHz, CDCl_3_): δ 209.4, 136.9, 134.5, 132.9, 128.5, 126.4, 126.2, 125.9, 123.9, 121.9, 120.7, 116.3, 107.6, 58.9, 49.9, 49.1, 30.4, 28.5, 20.7, 19.1, 9.2; FT—IR (ν, cm^- 1^, KBr): 3300 (N—H), 1681 (C=O).
Geometry. All e.s.d.'s (except the e.s.d. in the dihedral angle between two l.s. planes) are estimated using the full covariance matrix. The cell e.s.d.'s are taken into account individually in the estimation of e.s.d.'s in distances, angles and torsion angles; correlations between e.s.d.'s in cell parameters are only used when they are defined by crystal symmetry. An approximate (isotropic) treatment of cell e.s.d.'s is used for estimating e.s.d.'s involving l.s. planes.
Refinement. Refinement of *F*^2^ against ALL reflections. The weighted *R*-factor *wR* and goodness of fit *S* are based on *F*^2^, conventional *R*-factors *R* are based on *F*, with *F* set to zero for negative *F*^2^. The threshold expression of *F*^2^ > σ(*F*^2^) is used only for calculating *R*-factors(gt) *etc*. and is not relevant to the choice of reflections for refinement.

### Fractional atomic coordinates and isotropic or equivalent isotropic displacement parameters (Å^2^)

**Table:**

	*x*	*y*	*z*	*U*_iso_*/*U*_eq_	
O1	0.68329 (13)	0.4955 (3)	0.5734 (2)	0.0597 (10)	
N1	0.76542 (16)	0.5821 (4)	0.2820 (3)	0.0467 (10)	
H1	0.7453 (18)	0.563 (4)	0.204 (4)	0.056*	
C1	0.82420 (18)	0.6018 (4)	0.2844 (4)	0.0457 (12)	
C2	0.8445 (2)	0.6690 (4)	0.1988 (4)	0.0454 (12)	
C3	0.8088 (2)	0.7267 (5)	0.1164 (4)	0.0542 (14)	
H3	0.7691	0.7240	0.1190	0.065*	
C4	0.8307 (2)	0.7866 (5)	0.0329 (4)	0.0624 (15)	
H4	0.8064	0.8257	−0.0211	0.075*	
C5	0.8888 (3)	0.7892 (5)	0.0282 (5)	0.0674 (16)	
H5	0.9036	0.8278	−0.0316	0.081*	
C6	0.9245 (2)	0.7388 (5)	0.1057 (4)	0.0584 (15)	
H6	0.9641	0.7438	0.1009	0.070*	
C7	0.9041 (2)	0.6780 (5)	0.1950 (4)	0.0508 (13)	
C8	0.9406 (2)	0.6296 (5)	0.2821 (5)	0.0575 (14)	
H8	0.9802	0.6378	0.2817	0.069*	
C9	0.9198 (2)	0.5715 (5)	0.3664 (4)	0.0569 (13)	
H9	0.9449	0.5412	0.4251	0.068*	
C10	0.86094 (19)	0.5562 (4)	0.3669 (4)	0.0514 (13)	
H10	0.8468	0.5140	0.4250	0.062*	
C11	0.74053 (18)	0.5505 (4)	0.3731 (4)	0.0435 (12)	
H11	0.7592	0.5701	0.4439	0.052*	
C12	0.69145 (18)	0.4934 (4)	0.3731 (3)	0.0436 (12)	
C13	0.65587 (19)	0.4306 (4)	0.2821 (3)	0.0454 (12)	
H13	0.6557	0.4624	0.2051	0.054*	
C14	0.6757 (2)	0.3084 (4)	0.2953 (4)	0.0574 (14)	
H141	0.7173	0.3028	0.2978	0.069*	
H142	0.6583	0.2611	0.2336	0.069*	
C15	0.6547 (2)	0.2746 (5)	0.4094 (4)	0.0595 (14)	
H151	0.6869	0.2576	0.4654	0.071*	
H152	0.6297	0.2084	0.4008	0.071*	
C16	0.62158 (19)	0.3793 (4)	0.4453 (3)	0.0469 (12)	
C17	0.66827 (19)	0.4636 (4)	0.4773 (4)	0.0467 (13)	
C18	0.59773 (18)	0.4275 (4)	0.3300 (3)	0.0459 (12)	
C19	0.5713 (2)	0.5429 (5)	0.3385 (4)	0.0590 (14)	
H191	0.5393	0.5382	0.3836	0.088*	
H192	0.5581	0.5699	0.2634	0.088*	
H193	0.5995	0.5948	0.3738	0.088*	
C20	0.5540 (2)	0.3515 (5)	0.2652 (4)	0.0624 (15)	
H201	0.5440	0.3824	0.1903	0.094*	
H202	0.5202	0.3469	0.3051	0.094*	
H203	0.5700	0.2766	0.2586	0.094*	
C21	0.5823 (2)	0.3569 (6)	0.5342 (4)	0.0631 (15)	
H211	0.5603	0.4243	0.5460	0.095*	
H212	0.6044	0.3362	0.6042	0.095*	
H213	0.5566	0.2956	0.5099	0.095*	
O2	0.69132 (15)	0.5802 (3)	1.0744 (3)	0.0673 (11)	
N2	0.76421 (18)	0.5057 (4)	0.7714 (3)	0.0564 (12)	
H2	0.743 (2)	0.502 (5)	0.712 (4)	0.068*	
C31	0.8225 (2)	0.4882 (5)	0.7702 (4)	0.0512 (13)	
C32	0.8415 (2)	0.4213 (5)	0.6840 (4)	0.0554 (14)	
C33	0.8049 (2)	0.3591 (5)	0.6054 (4)	0.0588 (14)	
H33	0.7653	0.3617	0.6100	0.071*	
C34	0.8253 (3)	0.2968 (5)	0.5246 (5)	0.0674 (16)	
H34	0.7998	0.2569	0.4732	0.081*	
C35	0.8829 (3)	0.2900 (6)	0.5154 (5)	0.0750 (17)	
H35	0.8965	0.2475	0.4569	0.090*	
C36	0.9189 (3)	0.3432 (5)	0.5889 (5)	0.0700 (16)	
H36	0.9582	0.3376	0.5822	0.084*	
C37	0.8998 (2)	0.4094 (5)	0.6788 (5)	0.0572 (14)	
C38	0.9397 (2)	0.4604 (5)	0.7578 (5)	0.0621 (15)	
H38	0.9791	0.4515	0.7537	0.075*	
C39	0.9195 (2)	0.5234 (5)	0.8409 (5)	0.0708 (17)	
H39	0.9455	0.5581	0.8952	0.085*	
C40	0.8608 (2)	0.5376 (5)	0.8472 (4)	0.0582 (14)	
H40	0.8480	0.5817	0.9054	0.070*	
C41	0.7408 (2)	0.5355 (5)	0.8651 (4)	0.0575 (14)	
H41	0.7626	0.5197	0.9339	0.069*	
C42	0.68961 (18)	0.5857 (4)	0.8730 (4)	0.0452 (12)	
C43	0.6461 (2)	0.6346 (5)	0.7896 (4)	0.0547 (14)	
H43	0.6592	0.6503	0.7146	0.066*	
C44	0.5941 (2)	0.5569 (6)	0.7881 (4)	0.0674 (16)	
H441	0.5651	0.5764	0.7263	0.081*	
H442	0.6049	0.4775	0.7811	0.081*	
C45	0.5733 (2)	0.5810 (5)	0.9029 (4)	0.0621 (14)	
H451	0.5747	0.5127	0.9495	0.075*	
H452	0.5341	0.6097	0.8944	0.075*	
C46	0.6151 (2)	0.6717 (5)	0.9562 (4)	0.0524 (13)	
C47	0.6699 (2)	0.6078 (4)	0.9813 (4)	0.0458 (12)	
C48	0.62762 (19)	0.7388 (5)	0.8533 (4)	0.0556 (14)	
C49	0.6746 (2)	0.8251 (5)	0.8770 (5)	0.0661 (15)	
H491	0.6821	0.8616	0.8070	0.099*	
H492	0.7090	0.7877	0.9098	0.099*	
H493	0.6630	0.8812	0.9295	0.099*	
C50	0.5761 (2)	0.8006 (6)	0.7922 (5)	0.0802 (18)	
H501	0.5461	0.7466	0.7704	0.120*	
H502	0.5875	0.8377	0.7253	0.120*	
H503	0.5622	0.8565	0.8423	0.120*	
C51	0.5939 (3)	0.7335 (6)	1.0546 (4)	0.0796 (18)	
H511	0.6229	0.7860	1.0858	0.119*	
H512	0.5857	0.6796	1.1123	0.119*	
H513	0.5594	0.7747	1.0292	0.119*	

### Atomic displacement parameters (Å^2^)

**Table:**

	*U*^11^	*U*^22^	*U*^33^	*U*^12^	*U*^13^	*U*^23^
O1	0.059 (2)	0.080 (3)	0.0400 (18)	−0.0056 (19)	0.0018 (15)	−0.0112 (19)
N1	0.043 (2)	0.055 (3)	0.041 (2)	−0.004 (2)	−0.0013 (17)	0.000 (2)
C1	0.038 (3)	0.045 (3)	0.054 (3)	−0.002 (2)	0.004 (2)	−0.004 (2)
C2	0.051 (3)	0.041 (3)	0.046 (3)	−0.009 (3)	0.014 (2)	−0.011 (2)
C3	0.055 (3)	0.053 (3)	0.054 (3)	−0.014 (3)	0.007 (2)	−0.008 (3)
C4	0.076 (4)	0.063 (4)	0.048 (3)	−0.022 (3)	0.005 (3)	−0.002 (3)
C5	0.078 (4)	0.072 (4)	0.054 (3)	−0.037 (4)	0.019 (3)	−0.012 (3)
C6	0.055 (3)	0.063 (4)	0.061 (3)	−0.022 (3)	0.021 (3)	−0.017 (3)
C7	0.047 (3)	0.046 (3)	0.061 (3)	−0.008 (3)	0.010 (2)	−0.017 (3)
C8	0.040 (3)	0.049 (3)	0.085 (4)	−0.005 (3)	0.013 (3)	−0.015 (3)
C9	0.049 (3)	0.040 (3)	0.079 (3)	0.004 (3)	−0.005 (3)	−0.003 (3)
C10	0.047 (3)	0.043 (3)	0.065 (3)	−0.005 (3)	0.009 (2)	−0.003 (3)
C11	0.045 (3)	0.044 (3)	0.041 (2)	−0.001 (2)	0.002 (2)	−0.003 (2)
C12	0.040 (3)	0.050 (3)	0.041 (2)	−0.001 (2)	0.0016 (19)	0.001 (2)
C13	0.048 (3)	0.054 (3)	0.035 (2)	−0.007 (2)	0.0080 (19)	0.005 (2)
C14	0.064 (3)	0.057 (4)	0.053 (3)	−0.006 (3)	0.013 (2)	−0.007 (3)
C15	0.071 (3)	0.049 (3)	0.059 (3)	−0.006 (3)	0.008 (3)	0.004 (3)
C16	0.050 (3)	0.050 (3)	0.041 (2)	−0.008 (3)	0.007 (2)	0.001 (2)
C17	0.045 (3)	0.054 (3)	0.040 (3)	0.006 (2)	0.000 (2)	0.000 (2)
C18	0.045 (3)	0.051 (3)	0.041 (2)	−0.011 (2)	0.0043 (19)	−0.001 (2)
C19	0.047 (3)	0.066 (4)	0.063 (3)	−0.003 (3)	−0.001 (2)	0.002 (3)
C20	0.046 (3)	0.085 (4)	0.057 (3)	−0.024 (3)	0.009 (2)	−0.013 (3)
C21	0.063 (3)	0.078 (4)	0.051 (3)	−0.007 (3)	0.019 (2)	0.001 (3)
O2	0.077 (2)	0.076 (3)	0.0455 (18)	−0.020 (2)	−0.0127 (17)	0.0087 (19)
N2	0.049 (3)	0.064 (3)	0.055 (2)	0.006 (2)	0.0027 (19)	−0.001 (2)
C31	0.049 (3)	0.047 (3)	0.058 (3)	0.002 (3)	0.006 (2)	0.000 (3)
C32	0.054 (3)	0.040 (3)	0.072 (3)	0.005 (3)	0.007 (3)	0.017 (3)
C33	0.058 (3)	0.052 (4)	0.066 (3)	−0.003 (3)	0.004 (3)	0.009 (3)
C34	0.089 (5)	0.054 (4)	0.060 (3)	0.013 (3)	0.011 (3)	0.002 (3)
C35	0.095 (5)	0.065 (4)	0.066 (4)	0.010 (4)	0.013 (3)	0.006 (3)
C36	0.067 (4)	0.062 (4)	0.084 (4)	0.017 (3)	0.022 (3)	0.020 (4)
C37	0.048 (3)	0.045 (3)	0.079 (3)	0.005 (3)	0.009 (3)	0.017 (3)
C38	0.056 (3)	0.051 (4)	0.082 (4)	0.006 (3)	0.020 (3)	0.013 (3)
C39	0.058 (4)	0.051 (4)	0.101 (4)	−0.011 (3)	−0.004 (3)	0.015 (4)
C40	0.051 (3)	0.052 (3)	0.072 (3)	0.003 (3)	0.006 (3)	0.002 (3)
C41	0.061 (3)	0.063 (4)	0.048 (3)	−0.010 (3)	0.001 (2)	0.003 (3)
C42	0.039 (3)	0.053 (3)	0.043 (3)	0.004 (2)	0.003 (2)	0.000 (2)
C43	0.057 (3)	0.064 (4)	0.042 (3)	0.004 (3)	0.000 (2)	0.007 (3)
C44	0.056 (3)	0.083 (4)	0.060 (3)	0.013 (3)	−0.012 (2)	−0.004 (3)
C45	0.046 (3)	0.063 (4)	0.078 (3)	0.005 (3)	0.010 (3)	0.002 (3)
C46	0.055 (3)	0.053 (3)	0.051 (3)	0.004 (3)	0.015 (2)	0.001 (2)
C47	0.050 (3)	0.047 (3)	0.039 (3)	−0.004 (2)	−0.004 (2)	0.003 (2)
C48	0.050 (3)	0.058 (4)	0.060 (3)	0.011 (3)	0.007 (2)	0.012 (3)
C49	0.066 (3)	0.058 (4)	0.076 (3)	−0.002 (3)	0.016 (3)	0.009 (3)
C50	0.073 (4)	0.083 (5)	0.085 (4)	0.021 (4)	0.009 (3)	0.013 (4)
C51	0.093 (4)	0.077 (5)	0.073 (4)	0.002 (4)	0.031 (3)	−0.006 (3)

### Geometric parameters (Å, °)

**Table:**

O1—C17	1.235 (5)	O2—C47	1.229 (5)
N1—C11	1.351 (6)	N2—C41	1.353 (6)
N1—C1	1.416 (6)	N2—C31	1.404 (6)
N1—H1	1.03 (4)	N2—H2	0.83 (5)
C1—C10	1.370 (6)	C31—C40	1.368 (7)
C1—C2	1.427 (6)	C31—C32	1.419 (7)
C2—C3	1.419 (7)	C32—C37	1.404 (7)
C2—C7	1.429 (6)	C32—C33	1.430 (7)
C3—C4	1.378 (7)	C33—C34	1.353 (7)
C3—H3	0.9500	C33—H33	0.9500
C4—C5	1.391 (8)	C34—C35	1.390 (8)
C4—H4	0.9500	C34—H34	0.9500
C5—C6	1.341 (8)	C35—C36	1.331 (8)
C5—H5	0.9500	C35—H35	0.9500
C6—C7	1.421 (7)	C36—C37	1.449 (8)
C6—H6	0.9500	C36—H36	0.9500
C7—C8	1.416 (7)	C37—C38	1.413 (8)
C8—C9	1.362 (7)	C38—C39	1.375 (7)
C8—H8	0.9500	C38—H38	0.9500
C9—C10	1.414 (6)	C39—C40	1.418 (7)
C9—H9	0.9500	C39—H39	0.9500
C10—H10	0.9500	C40—H40	0.9500
C11—C12	1.353 (6)	C41—C42	1.371 (7)
C11—H11	0.9500	C41—H41	0.9500
C12—C17	1.462 (6)	C42—C47	1.451 (6)
C12—C13	1.516 (6)	C42—C43	1.488 (6)
C13—C14	1.541 (7)	C43—C44	1.547 (8)
C13—C18	1.551 (6)	C43—C48	1.551 (7)
C13—H13	1.0000	C43—H43	1.0000
C14—C15	1.559 (7)	C44—C45	1.540 (7)
C14—H141	0.9900	C44—H441	0.9900
C14—H142	0.9900	C44—H442	0.9900
C15—C16	1.563 (7)	C45—C46	1.566 (8)
C15—H151	0.9900	C45—H451	0.9900
C15—H152	0.9900	C45—H452	0.9900
C16—C21	1.512 (6)	C46—C47	1.516 (7)
C16—C17	1.522 (7)	C46—C51	1.523 (7)
C16—C18	1.555 (6)	C46—C48	1.528 (7)
C18—C19	1.526 (7)	C48—C49	1.529 (7)
C18—C20	1.535 (6)	C48—C50	1.553 (7)
C19—H191	0.9800	C49—H491	0.9800
C19—H192	0.9800	C49—H492	0.9800
C19—H193	0.9800	C49—H493	0.9800
C20—H201	0.9800	C50—H501	0.9800
C20—H202	0.9800	C50—H502	0.9800
C20—H203	0.9800	C50—H503	0.9800
C21—H211	0.9800	C51—H511	0.9800
C21—H212	0.9800	C51—H512	0.9800
C21—H213	0.9800	C51—H513	0.9800
			
C11—N1—C1	122.8 (4)	C41—N2—C31	122.4 (4)
C11—N1—H1	118 (3)	C41—N2—H2	117 (4)
C1—N1—H1	115 (2)	C31—N2—H2	120 (4)
C10—C1—N1	120.5 (4)	C40—C31—N2	121.5 (5)
C10—C1—C2	120.6 (4)	C40—C31—C32	119.9 (5)
N1—C1—C2	118.9 (4)	N2—C31—C32	118.6 (4)
C3—C2—C1	123.7 (4)	C37—C32—C31	118.6 (5)
C3—C2—C7	118.0 (5)	C37—C32—C33	117.4 (5)
C1—C2—C7	118.3 (5)	C31—C32—C33	124.0 (5)
C4—C3—C2	121.2 (5)	C34—C33—C32	121.5 (5)
C4—C3—H3	119.4	C34—C33—H33	119.2
C2—C3—H3	119.4	C32—C33—H33	119.2
C3—C4—C5	119.4 (5)	C33—C34—C35	121.1 (6)
C3—C4—H4	120.3	C33—C34—H34	119.4
C5—C4—H4	120.3	C35—C34—H34	119.4
C6—C5—C4	121.8 (5)	C36—C35—C34	119.7 (6)
C6—C5—H5	119.1	C36—C35—H35	120.1
C4—C5—H5	119.1	C34—C35—H35	120.1
C5—C6—C7	121.0 (5)	C35—C36—C37	121.9 (6)
C5—C6—H6	119.5	C35—C36—H36	119.1
C7—C6—H6	119.5	C37—C36—H36	119.1
C8—C7—C6	122.5 (5)	C32—C37—C38	121.9 (5)
C8—C7—C2	118.9 (5)	C32—C37—C36	118.2 (5)
C6—C7—C2	118.5 (5)	C38—C37—C36	119.9 (5)
C9—C8—C7	121.3 (5)	C39—C38—C37	117.7 (5)
C9—C8—H8	119.4	C39—C38—H38	121.1
C7—C8—H8	119.4	C37—C38—H38	121.1
C8—C9—C10	120.1 (5)	C38—C39—C40	121.5 (5)
C8—C9—H9	119.9	C38—C39—H39	119.3
C10—C9—H9	119.9	C40—C39—H39	119.3
C1—C10—C9	120.5 (5)	C31—C40—C39	120.5 (5)
C1—C10—H10	119.7	C31—C40—H40	119.8
C9—C10—H10	119.7	C39—C40—H40	119.8
N1—C11—C12	126.1 (4)	N2—C41—C42	128.0 (5)
N1—C11—H11	116.9	N2—C41—H41	116.0
C12—C11—H11	116.9	C42—C41—H41	116.0
C11—C12—C17	121.5 (4)	C41—C42—C47	120.8 (4)
C11—C12—C13	132.2 (4)	C41—C42—C43	133.6 (4)
C17—C12—C13	105.4 (4)	C47—C42—C43	105.5 (4)
C12—C13—C14	104.7 (4)	C42—C43—C44	105.9 (4)
C12—C13—C18	101.5 (3)	C42—C43—C48	101.3 (4)
C14—C13—C18	102.4 (4)	C44—C43—C48	102.9 (4)
C12—C13—H13	115.5	C42—C43—H43	115.0
C14—C13—H13	115.5	C44—C43—H43	115.0
C18—C13—H13	115.5	C48—C43—H43	115.0
C13—C14—C15	102.4 (4)	C45—C44—C43	101.8 (4)
C13—C14—H141	111.3	C45—C44—H441	111.4
C15—C14—H141	111.3	C43—C44—H441	111.4
C13—C14—H142	111.3	C45—C44—H442	111.4
C15—C14—H142	111.3	C43—C44—H442	111.4
H141—C14—H142	109.2	H441—C44—H442	109.3
C14—C15—C16	104.5 (4)	C44—C45—C46	104.4 (4)
C14—C15—H151	110.9	C44—C45—H451	110.9
C16—C15—H151	110.9	C46—C45—H451	110.9
C14—C15—H152	110.9	C44—C45—H452	110.9
C16—C15—H152	110.9	C46—C45—H452	110.9
H151—C15—H152	108.9	H451—C45—H452	108.9
C21—C16—C17	115.2 (4)	C47—C46—C51	115.8 (4)
C21—C16—C18	119.9 (4)	C47—C46—C48	101.1 (4)
C17—C16—C18	99.9 (4)	C51—C46—C48	118.6 (5)
C21—C16—C15	114.7 (5)	C47—C46—C45	103.4 (4)
C17—C16—C15	103.0 (4)	C51—C46—C45	114.1 (4)
C18—C16—C15	101.6 (4)	C48—C46—C45	101.6 (4)
O1—C17—C12	128.8 (5)	O2—C47—C42	128.7 (5)
O1—C17—C16	125.3 (4)	O2—C47—C46	126.1 (4)
C12—C17—C16	105.9 (4)	C42—C47—C46	105.2 (4)
C19—C18—C20	107.9 (4)	C46—C48—C49	113.7 (4)
C19—C18—C13	113.1 (4)	C46—C48—C43	93.7 (4)
C20—C18—C13	114.2 (4)	C49—C48—C43	113.4 (4)
C19—C18—C16	113.2 (4)	C46—C48—C50	115.1 (4)
C20—C18—C16	113.7 (4)	C49—C48—C50	107.2 (5)
C13—C18—C16	94.5 (3)	C43—C48—C50	113.5 (4)
C18—C19—H191	109.5	C48—C49—H491	109.5
C18—C19—H192	109.5	C48—C49—H492	109.5
H191—C19—H192	109.5	H491—C49—H492	109.5
C18—C19—H193	109.5	C48—C49—H493	109.5
H191—C19—H193	109.5	H491—C49—H493	109.5
H192—C19—H193	109.5	H492—C49—H493	109.5
C18—C20—H201	109.5	C48—C50—H501	109.5
C18—C20—H202	109.5	C48—C50—H502	109.5
H201—C20—H202	109.5	H501—C50—H502	109.5
C18—C20—H203	109.5	C48—C50—H503	109.5
H201—C20—H203	109.5	H501—C50—H503	109.5
H202—C20—H203	109.5	H502—C50—H503	109.5
C16—C21—H211	109.5	C46—C51—H511	109.5
C16—C21—H212	109.5	C46—C51—H512	109.5
H211—C21—H212	109.5	H511—C51—H512	109.5
C16—C21—H213	109.5	C46—C51—H513	109.5
H211—C21—H213	109.5	H511—C51—H513	109.5
H212—C21—H213	109.5	H512—C51—H513	109.5
			
C11—N1—C1—C10	21.3 (7)	C41—N2—C31—C40	−24.4 (8)
C11—N1—C1—C2	−159.3 (4)	C41—N2—C31—C32	158.1 (5)
C10—C1—C2—C3	−174.3 (5)	C40—C31—C32—C37	−1.3 (7)
N1—C1—C2—C3	6.2 (7)	N2—C31—C32—C37	176.1 (5)
C10—C1—C2—C7	6.2 (7)	C40—C31—C32—C33	174.7 (5)
N1—C1—C2—C7	−173.2 (4)	N2—C31—C32—C33	−7.8 (8)
C1—C2—C3—C4	−177.2 (5)	C37—C32—C33—C34	−4.1 (7)
C7—C2—C3—C4	2.2 (7)	C31—C32—C33—C34	179.9 (5)
C2—C3—C4—C5	0.9 (8)	C32—C33—C34—C35	0.5 (8)
C3—C4—C5—C6	−2.8 (9)	C33—C34—C35—C36	1.8 (9)
C4—C5—C6—C7	1.5 (9)	C34—C35—C36—C37	−0.4 (9)
C5—C6—C7—C8	−176.0 (5)	C31—C32—C37—C38	1.2 (8)
C5—C6—C7—C2	1.7 (8)	C33—C32—C37—C38	−175.2 (5)
C3—C2—C7—C8	174.3 (5)	C31—C32—C37—C36	−178.5 (5)
C1—C2—C7—C8	−6.2 (7)	C33—C32—C37—C36	5.2 (7)
C3—C2—C7—C6	−3.5 (7)	C35—C36—C37—C32	−3.1 (8)
C1—C2—C7—C6	176.0 (4)	C35—C36—C37—C38	177.2 (6)
C6—C7—C8—C9	−179.8 (5)	C32—C37—C38—C39	−0.3 (8)
C2—C7—C8—C9	2.5 (8)	C36—C37—C38—C39	179.3 (5)
C7—C8—C9—C10	1.5 (8)	C37—C38—C39—C40	−0.4 (8)
N1—C1—C10—C9	177.1 (5)	N2—C31—C40—C39	−176.7 (5)
C2—C1—C10—C9	−2.4 (8)	C32—C31—C40—C39	0.7 (8)
C8—C9—C10—C1	−1.6 (8)	C38—C39—C40—C31	0.2 (9)
C1—N1—C11—C12	−155.0 (5)	C31—N2—C41—C42	159.3 (5)
N1—C11—C12—C17	−179.9 (5)	N2—C41—C42—C47	177.4 (5)
N1—C11—C12—C13	12.9 (9)	N2—C41—C42—C43	−7.6 (10)
C11—C12—C13—C14	94.2 (6)	C41—C42—C43—C44	112.2 (6)
C17—C12—C13—C14	−74.5 (4)	C47—C42—C43—C44	−72.2 (5)
C11—C12—C13—C18	−159.5 (5)	C41—C42—C43—C48	−140.7 (6)
C17—C12—C13—C18	31.8 (5)	C47—C42—C43—C48	34.8 (5)
C12—C13—C14—C15	67.9 (4)	C42—C43—C44—C45	70.0 (5)
C18—C13—C14—C15	−37.7 (4)	C48—C43—C44—C45	−35.9 (5)
C13—C14—C15—C16	3.6 (5)	C43—C44—C45—C46	1.1 (5)
C14—C15—C16—C21	162.2 (4)	C44—C45—C46—C47	−70.1 (5)
C14—C15—C16—C17	−71.8 (4)	C44—C45—C46—C51	163.3 (5)
C14—C15—C16—C18	31.4 (5)	C44—C45—C46—C48	34.5 (5)
C11—C12—C17—O1	10.6 (8)	C41—C42—C47—O2	−5.9 (9)
C13—C12—C17—O1	−179.2 (5)	C43—C42—C47—O2	177.8 (5)
C11—C12—C17—C16	−167.4 (5)	C41—C42—C47—C46	175.3 (5)
C13—C12—C17—C16	2.8 (5)	C43—C42—C47—C46	−0.9 (6)
C21—C16—C17—O1	15.9 (7)	C51—C46—C47—O2	17.8 (8)
C18—C16—C17—O1	145.8 (5)	C48—C46—C47—O2	147.4 (5)
C15—C16—C17—O1	−109.7 (5)	C45—C46—C47—O2	−107.8 (6)
C21—C16—C17—C12	−166.0 (4)	C51—C46—C47—C42	−163.4 (5)
C18—C16—C17—C12	−36.1 (5)	C48—C46—C47—C42	−33.8 (5)
C15—C16—C17—C12	68.4 (4)	C45—C46—C47—C42	71.0 (5)
C12—C13—C18—C19	65.7 (5)	C47—C46—C48—C49	−65.4 (5)
C14—C13—C18—C19	173.7 (4)	C51—C46—C48—C49	62.4 (6)
C12—C13—C18—C20	−170.4 (4)	C45—C46—C48—C49	−171.7 (4)
C14—C13—C18—C20	−62.4 (5)	C47—C46—C48—C43	52.2 (4)
C12—C13—C18—C16	−51.9 (4)	C51—C46—C48—C43	179.9 (5)
C14—C13—C18—C16	56.2 (4)	C45—C46—C48—C43	−54.2 (4)
C21—C16—C18—C19	62.3 (6)	C47—C46—C48—C50	170.3 (5)
C17—C16—C18—C19	−64.5 (4)	C51—C46—C48—C50	−61.9 (7)
C15—C16—C18—C19	−170.2 (4)	C45—C46—C48—C50	64.0 (6)
C21—C16—C18—C20	−61.2 (6)	C42—C43—C48—C46	−53.2 (4)
C17—C16—C18—C20	171.9 (4)	C44—C43—C48—C46	56.2 (4)
C15—C16—C18—C20	66.3 (5)	C42—C43—C48—C49	64.7 (5)
C21—C16—C18—C13	179.8 (5)	C44—C43—C48—C49	174.1 (4)
C17—C16—C18—C13	53.0 (4)	C42—C43—C48—C50	−172.7 (4)
C15—C16—C18—C13	−52.7 (4)	C44—C43—C48—C50	−63.3 (5)

### Hydrogen-bond geometry (Å, °)

**Table:**

*D*—H···*A*	*D*—H	H···*A*	*D*···*A*	*D*—H···*A*
N1—H1···O2^i^	1.03 (4)	1.93 (4)	2.909 (5)	157 (4)
N2—H2···O1	0.83 (5)	2.08 (5)	2.913 (5)	174 (5)

Symmetry codes: (i) *x*, *y*, *z*−1.
